# A Cholera Conjugate Vaccine Containing O-specific Polysaccharide (OSP) of *V. cholerae* O1 Inaba and Recombinant Fragment of Tetanus Toxin Heavy Chain (OSP:rTTHc) Induces Serum, Memory and Lamina Proprial Responses against OSP and Is Protective in Mice

**DOI:** 10.1371/journal.pntd.0003881

**Published:** 2015-07-08

**Authors:** Md. Abu Sayeed, Meagan Kelly Bufano, Peng Xu, Grace Eckhoff, Richelle C. Charles, Mohammad Murshid Alam, Tania Sultana, Md. Rasheduzzaman Rashu, Amanda Berger, Geoffrey Gonzalez-Escobedo, Anjali Mandlik, Taufiqur Rahman Bhuiyan, Daniel T. Leung, Regina C. LaRocque, Jason B. Harris, Stephen B. Calderwood, Firdausi Qadri, W. F. Vann, Pavol Kováč, Edward T. Ryan

**Affiliations:** 1 Division of Infectious Diseases, Massachusetts General Hospital, Boston, Massachusetts, United States of America; 2 Center for Vaccine Sciences, International Centre for Diarrhoeal Disease Research Bangladesh (icddr,b), Dhaka, Bangladesh; 3 National Institute of Diabetes, Digestive and Kidney Diseases (NIDDK), Laboratory of Bioorganic Chemistry (LBC), National Institutes of Health, Bethesda, Maryland, United States of America; 4 Harvard Medical School, Boston, Massachusetts, United States of America; 5 Division of Infectious Disease, University of Utah School of Medicine, Salt Lake City, Utah, United States of America; 6 Department of Pediatrics, Harvard Medical School, Boston, Massachusetts, United States of America; 7 Department of Microbiology and Immunobiology, Harvard Medical School, Boston, Massachusetts, United States of America; 8 Center for Biologics Evaluation and Research (CBER), Food and Drug Administration (FDA), Laboratory of Bacterial Toxins, Bethesda, Maryland, United States of America; 9 Department of Immunology and Infectious Disease, Harvard School of Public Health, Boston, Massachusetts, United States of America; University of Tennessee, UNITED STATES

## Abstract

**Background:**

*Vibrio cholerae* is the cause of cholera, a severe watery diarrhea. Protection against cholera is serogroup specific. Serogroup specificity is defined by the O-specific polysaccharide (OSP) component of lipopolysaccharide (LPS).

**Methodology:**

Here we describe a conjugate vaccine for cholera prepared via squaric acid chemistry from the OSP of *V*. *cholerae* O1 Inaba strain PIC018 and a recombinant heavy chain fragment of tetanus toxin (OSP:rTTHc). We assessed a range of vaccine doses based on the OSP content of the vaccine (10-50 μg), vaccine compositions varying by molar loading ratio of OSP to rTTHc (3:1, 5:1, 10:1), effect of an adjuvant, and route of immunization.

**Principle Findings:**

Immunized mice developed prominent anti-OSP and anti-TT serum IgG responses, as well as vibriocidal antibody and memory B cell responses following intramuscular or intradermal vaccination. Mice did not develop anti-squarate responses. Intestinal lamina proprial IgA responses targeting OSP occurred following intradermal vaccination. In general, we found comparable immune responses in mice immunized with these variations, although memory B cell and vibriocidal responses were blunted in mice receiving the highest dose of vaccine (50 μg). We found no appreciable change in immune responses when the conjugate vaccine was administered in the presence or absence of immunoadjuvant alum. Administration of OSP:rTTHc resulted in 55% protective efficacy in a mouse survival cholera challenge model.

**Conclusion:**

We report development of an Inaba OSP:rTTHc conjugate vaccine that induces memory responses and protection against cholera in mice. Development of an effective cholera conjugate vaccine that induces high level and long-term immune responses against OSP would be beneficial, especially in young children who respond poorly to polysaccharide antigens.

## Author Summary

Cholera is a severely dehydrating diarrheal disease that remains an important global public health problem especially in developing countries. Globally, almost all cases of cholera are caused by *V*. *cholerae* O1. *V*. *cholerae* O1 organisms can be classified into two serotypes, Ogawa and Inaba, based on the presence or absence of a methyl group on the upstream terminal moiety of OSP. Immunity against cholera is serogroup-specific and serogroup specificity is determined by the O-specific polysaccharide (OSP) of *V*. *cholerae* lipopolysaccharide (LPS). Previous infection with Ogawa provides protection against subsequent Ogawa infection, while previous infection with Inaba provides more complete protection against subsequent Inaba and Ogawa infection. We have previously reported development of an Ogawa cholera conjugate vaccine. Here we report development of an Inaba OSP:rTTHc cholera conjugate vaccine and assessment of a range of doses, molar ratios of polysaccharide to carrier, immunization routes, and presence or absence of adjuvant.

## Introduction

Cholera is a severely dehydrating diarrheal disease caused by toxigenic strains of *Vibrio cholerae*. Cholera spreads through fecal-oral transmission and is closely associated with poor sanitation and lack of safe drinking water. Cholera remains an important global public health problem, especially in areas of Asia, Africa, and the Caribbean [1,2]. Globally, approximately 3–5 million cases of cholera and 100,000–130,000 deaths due to cholera occur each year [3]. Children are at a particularly high risk for cholera in endemic areas [3]. In these areas, children who are younger than 5 years of age have a 2–4 times higher incidence rate of cholera than those of the overall population [1,2,4,5,6,7]. Vaccination against cholera can assist in control measures until safe water and adequate sanitation become a reality for the impoverished and disenfranchised who are most at risk for cholera [8].

There are more than 200 serogroups of *V*. *cholerae*, classified based on the O-specific polysaccharide (OSP) antigen of lipopolysaccharide (LPS) [1]. Among these serogroups, cholera is mainly caused by *V*. *cholerae* serogroup O1, and less commonly by *V*. *cholerae* serogroup O139 [3,6]. Protection against cholera is serogroup specific. Previous infection with *V*. *cholerae* O1 does not provide protection against O139, and vice versa [9,10,11]. Globally, almost all cases of cholera are caused by *V*. *cholerae* O1. *V*. *cholerae* O1 organisms can be classified into two serotypes, Ogawa and Inaba, based, respectively, on the presence or absence of a methyl group on the upstream terminal moiety of OSP [12]. Previous infection with Ogawa provides protection against subsequent Ogawa infection, while previous infection with Inaba provides more complete protection against subsequent Inaba and Ogawa infection [13,14,15]. We have previously reported development of an Ogawa cholera conjugate vaccine [16]. Here we report development of an Inaba cholera conjugate vaccine and assessment of a range of doses, molar ratios of polysaccharide to carrier, immunization routes, and presence or absence of adjuvant.

## Materials and Methods

### Ethics statement

Animal work met all governmental and institutional requirements, guidelines, and policies. This work was approved by the Massachusetts General Hospital Subcommittee on Research Animal Care (SRAC). The work adheres to the USDA Animal Welfare Act, PHS Policy on Humane Care and Use of Laboratory Animals, and the ‘‘ILAR Guide for the Care and Use of Laboratory Animals”. Serum from humans recovering from cholera and typhoid were collected from patients at the International Centre for Diarrhoeal Disease Research in Dhaka, Bangladesh (icddr,b). This study was approved by the Ethical Review and Research Review Committee of the icddr,b and the Institutional Review Board of Massachusetts General Hospital. Written informed consent was obtained from guardians of child participants (<18 years), and adult participants (≥18 years) provided their own consent.

### Study population

We enrolled patients 2–50 years of age hospitalized at the Dhaka Hospital of the icddr,b who presented with severe acute watery diarrhea and stool culture positive for *V*. *cholerae* O1 (cholera; n = 5), or ≥3 days of fever ≥39°C and a positive blood culture for *Salmonella enterica* serovar Typhi (typhoid fever; n = 5). After informed consent of patients or parents/guardians, we obtained blood by venipuncture on days 0–2 (acute stage) and 7–21 (convalescence).

### Bacterial strains and media

*V*. *cholerae* O1 El Tor Inaba strain PIC018 isolated in 2007 from a patient with cholera in Bangladesh was used to prepare OSP for use in vaccine preparation and for immunological assays. *V*. *cholerae* O1 El Tor Inaba strain N16961 was used in the mouse neonatal challenge assay. Strains were grown in Luria-Bertani (LB) broth or on LB agar plates.

### General methods for production and analysis of conjugates

Optical rotations were measured at ambient temperature for solution in CHCl_3_ with a Perkin–Elmer automatic polarimeter, Model 341. Melting points were measured on a Kofler hot stage. All reactions were monitored by thin-layer chromatography (TLC) on silica gel 60 coated glass slides. Spots were visualized by charring with 5% H_2_SO_4_ in ethanol, 1% ninhydrine in ethanol, or UV light, as required. Column chromatography was performed by elution from prepacked (Biotage, Inc.) columns of silica gel with a Isolera Flash Chromatograph (Biotage), the latter being connected to an external Evaporative Light Scattering Detector, Model 380-LC (Varian, Inc.). Nuclear Magnetic Resonance (NMR) spectra were measured at 600 MHz for ^1^H, and 150 MHz for ^13^C with Bruker Avance spectrometers. Solvent peaks were used as internal references relative to TMS for ^1^H and ^13^C chemical shifts (ppm). Assignments of NMR signals were made by homonuclear and heteronuclear two-dimensional correlation spectroscopy, run with software supplied with the spectrometers. Liquid Chromatography–Electron Spray-Ionization Mass Spectrometry (ESI-MS) was performed with a Hewlett–Packard 1100 MSD spectrometer. Solutions in organic solvents were dried with anhydrous Na_2_SO_4_, and concentrated at 40°C/2 kPa.

### Vaccine preparation

LPS was isolated from *V*. *cholerae* O1 El Tor Inaba strain PIC018 and OSP-core was derived from LPS as previously described [16,17,18,19], with an additional filtering step to recover OSP product between 3 and 30 kDa. Recombinant tetanus toxoid heavy chain fragment (rTTHc) was prepared as a 52,108 Da recombinant protein in *E*. *coli* BL21 (DE3) as previously described [16]. Conjugation of OSP to rTTHc was performed via squaric acid chemistry as previously described [16,19]. Briefly, 3,4-dimethoxy-3-cyclobutene-1, 2-dione (dimethyl squarate, Sigma/Aldrich) was added to a solution of Inaba O-SP–core antigen in pH 7 phosphate buffer (0.05 M), and the mixture was gently stirred at room temperature for 48 hours. The solution was then transferred into an Amicon Ultra (3K cutoff) ultrafiltration tube and dialyzed against pure water (centrifugation at 4°C, 7,500× g, 8 times, 35 min each time). The retentate was lyophilized to afford the O-SP–core squarate monomethyl ester as a white solid.

rTTHc and the methyl squarate derivative of the Inaba O-SP–core antigen were mixed in 0.5 M pH 9 borate buffer. The mixture was stirred at room temperature and the progress of the reaction was monitored by SELDI-TOF MS [20]. OSP:rTTHc products were generated with molar ratios of OSP to rTTHc of 3:1, 5:1, and 10:1. The mixtures were transferred into an Amicon Ultra (30 K cutoff) centrifuge tube and dialyzed (centrifugation at 4°C, 7,500× g, 8 times, 6 min each time) against 10 mM aqueous ammonium carbonate and then lyophilized. The average MW of the OSP antigen was 5,900 Da. A corresponding conjugate (OSP:BSA ratio: 5:1) for use in immunological assays was made of OSP and bovine serum albumin (BSA; Sigma), purified as previously described [21]. Alum (Pierce Biotechnology) was used as the immunoadjuvant in a 1:1 dilution with conjugate vaccine or saline control.

### Preparation of a squarate-labeled synthetic conjugate 3

To assess antibody responses potentially targeting squarate and not OSP, we also produced a synthetic conjugate **3** from a short, unrelated, squarate moiety-containing linker construct **2** and BSA (squarate to BSA molar ratio: 6:1). Preparation of linker **1** was previously briefly described [22], but not characterized. It was now obtained in crystalline condition and fully characterized. A squarate-labeled construct **2**, also crystalline and obtained in analytically pure condition, was synthesized for the first time as described below; its identity was established by ^1^H and ^13^C spectroscopy.

### Preparation of squarate-labeled construct 2

#### N-(2-aminoethyl)-6-hydroxyhexanamide (1)

In a pear shaped 50 mL flask, ε-caprolactone (1.0 g, 8.76 mmol) was treated at room temperature with ethylenediamine (17.7 mL, 263 mmol) for 18 h. Thin-layer chromatography (TLC) showed disappearance of ε-caprolactone and formation of a more polar ninhydrine-positive material. After concentration at 30°C, to remove most of the ethylenediamine, the residue was chromatographed (1:5:0.1 EtOAc–MeOH–25% aq. NH_3_). *N*-(2-aminoethyl)-6-hydroxyhexanamide **1** (1.46 g, 8.38 mmol) was obtained as syrup (96%), which crystallized readily from 2-propanol–hexane, m.p. 1.5–63°C, after drying at 40°C/133 Pa overnight; ^1^H-NMR (600 MHz, DMSO): δ 7.73 (s, 1H,-NH-), 3.36 (t, *J* = 6.6 Hz, 2H, H-6), 3.01 (q, J = 6.4 Hz, 2H, H-1′), 2.52 (t, *J* = 6.5 Hz, 2H, H-2′), 2.04 (t, *J* = 7.5 Hz, 2H, H-2), 1.47 (m, 2H, H-3), 1.39 (m, 2H, H-5), 1.24 (m, 2H, H-4); ^13^C-NMR (150 MHz, DMSO): δ 172.2 (C-1), 60.6 (C-6), 42.2 (C-1′), 41.4 (C-2′), 35.5 (C-2), 32.3 (C-5), 25.3 (C-3, C-4); TOF-HRMS *m/z*: [M+H]^+^ Calculated for C_8_H_19_N_2_O_2_: 175.1441, Found 175.1444. The analytical sample was dried at 80°C/133 Pa for 2 h and allowed to cool to room temperature, whereupon the material solidified. Analytic Calculated for 3 C_x_H_z_N_w_.H_2_O: C, 53.31; H, 10.44; N, 15.54. Found: C, 53.17; H, 10.17; N, 15.69. The compound appeared polymorphous. Crystallization from EtOH–EtOAc yielded material melting at 71–75°C.

#### 6-Hydroxy-N-[2-(2-methoxycyclobutene-3,4-dione-1)aminoethyl]hexanamide (2)

A solution of compound 1 (500 mg, 2.87 mmol) and 3,4-dimethoxy-3-cyclobutene-1,2-dione (489 mg, 3.44 mmol) in methanol (MeOH) was kept at room temperature for 18 h, when TLC showed that a faster moving, ultraviolet-positive product was formed. The mixture was concentrated, and chromatography 15:1 → 10:1 CH_2_Cl_2_–MeOH gave squarate-labeled linker **2** (735 mg, 2.59 mmol, 90%) as syrup, which solidified on standing. After crystallization from MeOH, white crystal obtained melted at 105–106.5°C; ^1^H-NMR (600 MHz, D_2_O): δ 4.39 and 4.36 (2s, 3H, OCH_3_), 3.72 and 3.59 (2m, 2H, H-2′), 3.58 (t, *J* = 6.5 Hz, 2H, H-6), 3.42 (m, 2H, H-1′), 2.27–2.19 (m, 2H, H-2), 1.61–1.49 (m, 4H, H-3, H-5), 1.35–1.27 (m, 2H, H-4); ^13^C-NMR (150 MHz, D_2_O): δ 178.4 (C-1), 62.1 (C-6), 61.6 (OCH_3_), 44.7 and 44.6 (C-2′), 40.1 and 39.9 (C-1′), 36.4 (C-2), 31.6 (C-5), 25.8 and 25.7 (C-3), 25.3 and 25.2 (C-4); TOF-HRMS *m/z*: [M+H]^+^ Calculated for C_13_H_21_N_2_O_5_: 285.2450, Found 285.1449; Analytic Calculated for C_13_H_20_N_2_O_5_: C, 54.92; H, 7.09; N, 9.85. Found: C, 54.72; H, 6.90; N, 9.64.

### Assessment of conjugates using human serum

To assess immunoreactivity of the OSP display on the conjugates, we performed antigen-specific ELISAs using sera collected from humans with cholera in Bangladesh, and compared these responses to those detected in humans with typhoid fever in Bangladesh. We coated the wells with either OSP:rTTHc (5:1; 100 ng/well), OSP:BSA (100 ng/well), or TT (Astarte; 100 ng/well). Following blocking and washing of plates, we added acute and convalescent phase sera from humans with cholera or typhoid (diluted 1:500 in 0.1% BSA in phosphate buffered saline-Tween). The presence of antigen-specific antibodies was detected using horseradish peroxidase conjugated to anti-human IgG antibody (diluted 1:5000 in 0.1% BSA in phosphate buffered saline-Tween) (Jackson ImmunoResearch). After 1.5 h incubation at 37°C, we developed the plates with a 0.55 mg/ml solution of 2,2ʹ 0-azinobis (3-ethylbenzothiazoline-6-sulfonic acid) (ABTS; Sigma) with 0.03% H_2_O_2_ (Sigma), and determined the optical density at 405 nm with a Vmax microplate kinetic reader (Molecular Devices Corp., Sunnyvale, CA). Plates were read for 5 min at 30 s intervals, and the maximum slope for an optical density change of 0.2 U was reported as millioptical density units per minute (mOD/min).

### Immunization and collection of samples

To assess immunogenicity of the OSP:rTTHc conjugate vaccine following immunization, we used 10 cohorts (n = 10–15) of female Swiss-Webster (3–5 week old) mice. We intramuscularly immunized one cohort of mice with 5:1 molar ratio of OSP to rTTHc vaccine (10 μg OSP per mouse) without alum, 3 cohorts of mice (10 μg of OSP per mouse) at different molar ratios of vaccines (OSP:rTTHc ratio of 3:1, 5:1, 10:1) with alum, and 2 cohorts of mice with 5:1 molar ratio of OSP to rTTHc vaccine at different doses (20 μg and 50 μg of OSP per mouse) with alum. We also immunized 2 cohorts of mice intradermally with 5:1 molar ratio of OSP to rTTHc vaccine (10 μg OSP per mouse), with or without alum. As additional control groups, we used 2 cohorts of mice who received alum only, either intramuscularly or intradermally. Mice were immunized on days 0, 21, and 42. We collected blood samples via tail bleeding on days 0, 7, 21, 28, 42, 49 and 56. Samples were collected, processed, aliquoted, and stored as previously described [18,23]. For the memory B cell assay, splenocytes were isolated after day 56 and processed for ELISPOT as previously described [16]. Lamina proprial tissue was also collected after day 56 and processed to measure antigen-specific IgA secreting cell responses, as described below. For use in the *V*. *cholerae* wild type challenge assay, sera were collected from additional cohorts of mice immunized with 10 μg OSP per mouse (OSP:rTTHc; 5:1) with alum, or alum alone on days 0, 14 and 28.

### Assessment of anti-squarate responses

To assess whether antibodies targeting the squarate moiety occur following immunization with OSP:rTTHc, we coated ELISA plate wells with OSP-squarate-BSA (100 ng/well) or squarate-BSA (100 ng/well; synthetic conjugate 3, see above) and assessed antigen-specific IgG responses as described below.

### Antigen-specific antibody responses in serum

OSP and TT-specific IgG, IgM and IgA responses in serum were measured using standard enzyme-linked immunosorbent assay (ELISA) protocols as previously described [16,18,23]. Briefly, to assess anti-OSP and anti-TT antibody responses, ELISA plates were coated with *V*. *cholerae* O1 Inaba OSP:BSA (1 μg/mL) or TT (1 μg/mL) in 50 mM carbonate buffer [16,17]. Sera (diluted 1:25 in 0.1% BSA in phosphate buffered saline-Tween) were added at 100 μl per well and the presence of antigen-specific antibodies was detected using horseradish peroxidase-conjugated to anti-mouse IgG, IgM or IgA antibody (diluted 1:1000 in 0.1% BSA in phosphate buffered saline-Tween) (Southern Biotech). Plates were developed and read as described above. We defined a responder as having more than a 15-times increase of ELISA Units for OSP-specific IgG responses compared with baseline day 0 levels, and a 30-times increase for TT-specific IgG responses when compared to baseline levels.

### Serum vibriocidal responses

We assessed serum vibriocidal antibody titers against *V*. *cholerae* O1 El Tor Inaba strain PIC018 in a micro-assay as previously described [16,24,25]. We inactivated endogenous complement activity of mouse serum by heating it for 1 hr at 56°C. We then added 50 μl aliquots of two-fold serial dilutions of heat-inactivated sera in 0.15 M saline (1:1 to 1:1024) to wells of sterile 96-well tissue culture plates containing 50 μl/well of *V*. *cholerae* O1 El Tor Inaba strain PIC018 (OD 0.1) in 0.15 M saline and 22% guinea pig complement (EMD Biosciences). Plates were then incubated for 1 hr at 37°C. 150 μl of brain heart infusion broth (Becton Dickinson) was added to each well, and plates were incubated for an additional 1.5 h at 37°C, when optical density at 595 nm was assessed. We calculated the vibriocidal titer as the dilution of serum causing 50% reduction in optical density compared with that of wells containing no serum [26,27]. We defined a responder as having at least a 4-fold increase of vibriocidal titer at day 56 compared with baseline day 0 titer.

### Memory B cell responses

We assessed memory B cell responses after the third round of immunization as previously described [16,28]. Briefly, we treated splenocytes from mice with 1 ml erythrocyte lysis buffer (Sigma) and re-suspended them in RPMI-1640 supplemented with 10% fetal bovine serum (FBS) (Hyclone), betamercaptoethanol (Sigma), R595 LPS (Alexis), ConA stimulated supernatant and antibiotics (penicillin, streptomycin). The ConA-stimulated supernatant was made from naive mice splenocytes cultured with 2.5 μg/ml ConA and 20 ng/ml PMA for 48 hours at 37°C in a humid atmosphere with 5% CO_2_. We then cultured spleen cells in 96 well round-bottom plates containing 1×10^7^ cells/ml irradiated syngeneic spleen cell feeders (1200 rad) from naive mice, and 1×10^5^ cells/well from immunized mice in a total volume of 200 μl. Plates were then incubated at 37°C in a humid atmosphere with 5% CO_2_. After 6 days of culture, cells were harvested and antigen-specific memory B cell responses were measured by conventional ELISPOT method. We assessed total IgA and IgG secreting cells as well as OSP and TT-specific cells by ELISPOT assay using these cultured cells. Specifically, nitrocellulose bottom plates (MAHAS4510, Millipore) were coated with OSP:BSA (100 ng/well), or TT (100 ng/well), goat anti-mouse IgA (Southern Biotech), goat anti-mouse IgG (Southern Biotech), or keyhole limpet hemocyanin (KLH; Pierce Biotechnology) (2.5 μg/ml, negative control). After blocking the plates with RPMI-1640 supplemented with 10% FBS, we added the cultured cells to the wells and incubated the plates for 5 h at 37°C in a humid atmosphere with 5% CO_2_. We then added biotinylated anti-mouse IgA or IgG (Southern Biotech) antibody diluted at 1:1000 in PBS-Tween-FBS followed by the addition of horseradish peroxidase-conjugated avidin-D (5 mg/ml, Vector Labs) diluted at 1:1000 in PBS-Tween-FBS to detect IgA or IgG antibody secreting cells. Plates were developed with AEC (3 amino-9-ethyl-carbozole; Sigma). We used unstimulated samples as negative controls and assessed responses to KLH as a control for the plate. We excluded plates having more than 3 KLH spots. We defined a responder as having more than 5 times total IgG secreting cells with stimulation versus no stimulation, and >3.5 anti-OSP spots per 10^5^ splenocytes for OSP-specific memory B cell responses, or >5 anti-TT spots per 10^5^ splenocytes for TT-specific memory B cell responses.

### Lamina proprial IgA secreting lymphocyte responses

We euthanized mice after day 56 and collected the whole small intestines in HBSS media (Hank’s Balanced Salt Solution; Hyclone) supplemented with 4% fetal bovine serum (FBS, Invitrogen) and 1% HEPES (Invitrogen), storing at 4°C until processing. We then removed fat and collected any evident Peyer’s Patches, dissecting under magnification. We then removed residual fecal content and washed the intestine once with HBSS. Subsequently the intestine was cut into small pieces and washed three times with media. These pieces were incubated with 0.1 mM EDTA in HBSS for 25 mins at 37°C with stirring, and the supernatant was aspirated in order to remove intestinal epithelial cells. Residua was washed twice with RPMI-1640 Complete Media containing L-glutamine (Invitrogen) and supplemented with heat-inactivated 5% FBS, 1% penicillin-streptomycin and 1% Fungizone (Invitrogen). Residua was then incubated with collagenase in RPMI Complete Media for 30–35 min at 37°C. The cell suspension was collected using a 70 μm strainer (Becton Dickinson) and the residua was treated with collagenase again. The cell suspensions were pooled and washed with RPMI-1640 Complete Media containing L-glutamine (Invitrogen) supplemented with heat-inactivated 10% FBS, 1% penicillin-streptomycin and 1% Fungizone (Invitrogen). Lymphocytes were then isolated by density gradient centrifugation method using Lympholyte-M (Cedarlane). Antigen-specific IgA and total IgA secreting lymphocyte responses were then measured by conventional ELISPOT method. Specifically, nitrocellulose bottom plates (MAHAS4510, Millipore) were coated with OSP:BSA (100 ng/well) or with goat anti-mouse IgA (Southern Biotech) or with keyhole limpet hemocyanin (KLH; Pierce Biotechnology) (1 μg/mL, negative control). After we blocked the plates with RPMI supplemented with 10% FBS, we added the lymphocytes to the wells and incubated the plates for 5 h at 37°C in a humid atmosphere with 5% CO_2_. We then added biotinylated anti-mouse IgA (Southern Biotech) antibody at 1:1000, detected IgA antibody secreting cells using horseradish peroxidase-conjugated avidin-D (5 mg/ml, Vector Labs), and developed plates with AEC. We excluded plates having more than 3 KLH spots. We defined a responder as having >3 anti-OSP spots per 10^6^ lymphocytes.

### Neonatal challenge experiments

To assess protection afforded by immunization, we used a cholera neonatal mouse challenge assay, as previously described [16,18,23], using wild-type *V*. *cholerae* O1 El Tor Inaba strain N16961. In brief, we removed three to five day old un-immunized CD-1 suckling mice from dams two hours prior to inoculation. We then administered to pups a 50 μl inoculum comprised of 5.8x10^9^cfu of *V*. *cholerae* O1 El Tor Inaba strain N16961 mixed with a 1:250 dilution of pooled day 42 sera from mice intramuscularly immunized on days 0, 14, and 28 with OSP:TThc (5:1; 10 μg of OSP per dose) with alum or immunized with alum alone. Following oral challenge, we kept neonates at 30°C and monitored animals every 3 hr for 36 hr, after which surviving animals were euthanized.

### Statistics and graphs

We compared data from different groups using Mann-Whitney U tests. Within each group, comparisons of data from different time points to baseline data (day 0) were carried out using Wilcoxon Signed-Rank tests. Kaplan-Meier and log rank analysis were performed to compare survival curves in the neonatal challenge study. All reported P values were two-tailed, with a cutoff of *P*< 0.05 considered a threshold for statistical significance. We performed statistical analyses using GraphPad Prism 4 (GraphPad Software, Inc.).

## Results

### Immunoreactivity of OSP-conjugates using sera from humans with cholera in Bangladesh

**Fig 1:**
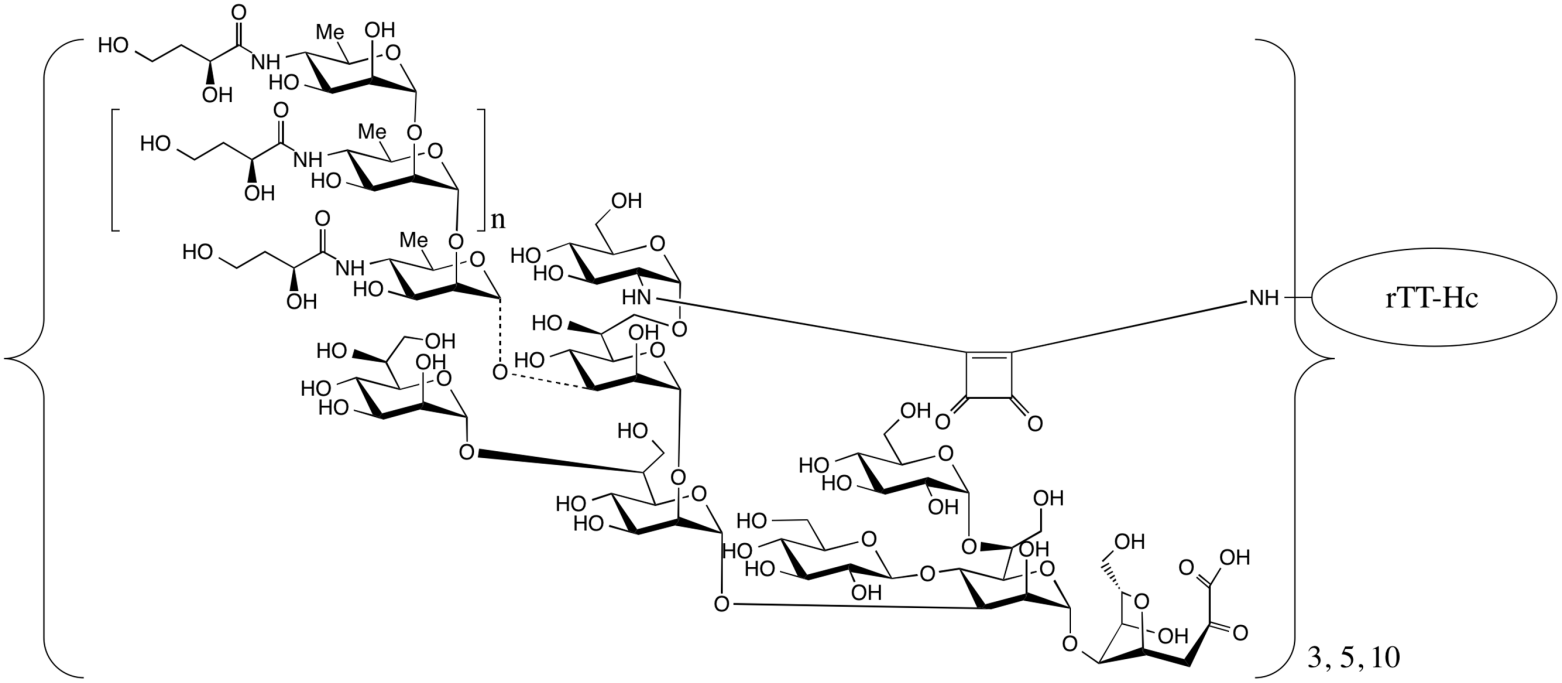
Structure of Inaba OSP:rTTHc conjugate. Variations of conjugates containing 3, 5, or 10 moles of OSP per mole carrier were developed. rTTHc = recombinant tetanus toxoid heavy chain fragment.

**Fig 2:**
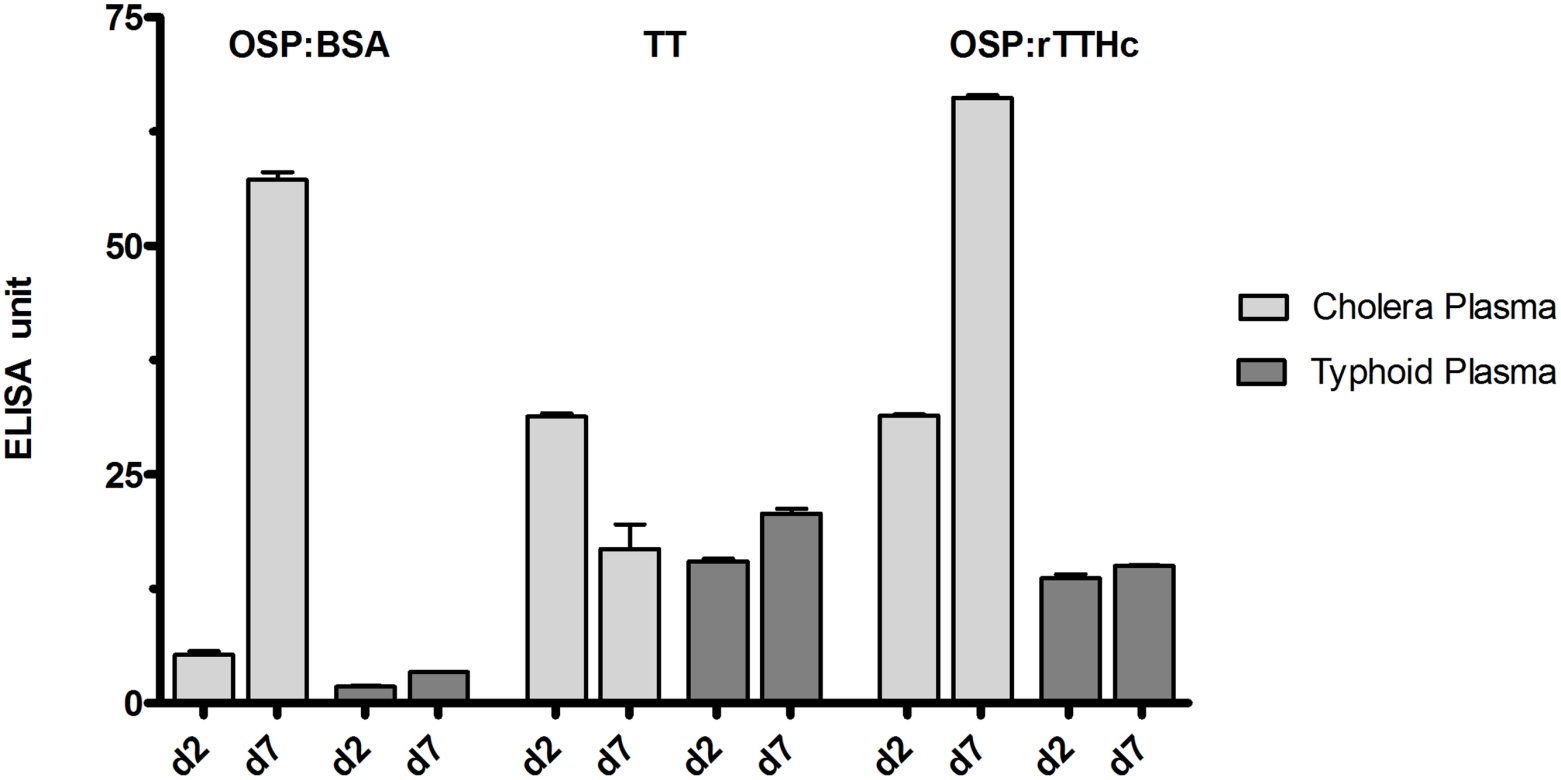
Immunoreactivity in human plasma of OSP:rTTHc, OSP:BSA, and TT. Immunoreactivity of OSP:rTTHc, OSP:BSA and TT was measured in day 2 versus day 7 plasma of patients with cholera versus typhoid fever in Dhaka, Bangladesh.

To assess whether the conjugates displayed OSP in a immunologically relevant manner, we assessed immunoreactivity of OSP:rTTHc (Fig 1) and OSP:BSA using sera from humans with cholera or typhoid in Bangladesh. We found a significant increase in immunoreactivity to these conjugates in humans recovering from cholera, but not typhoid (Fig 2). As expected, we found no change in antibody levels to tetanus toxoid during the course of clinical illness in either group of patients.

### OSP and TThc-specific serum antibody and vibriocidal responses in vaccinated animals

**Fig 3:**
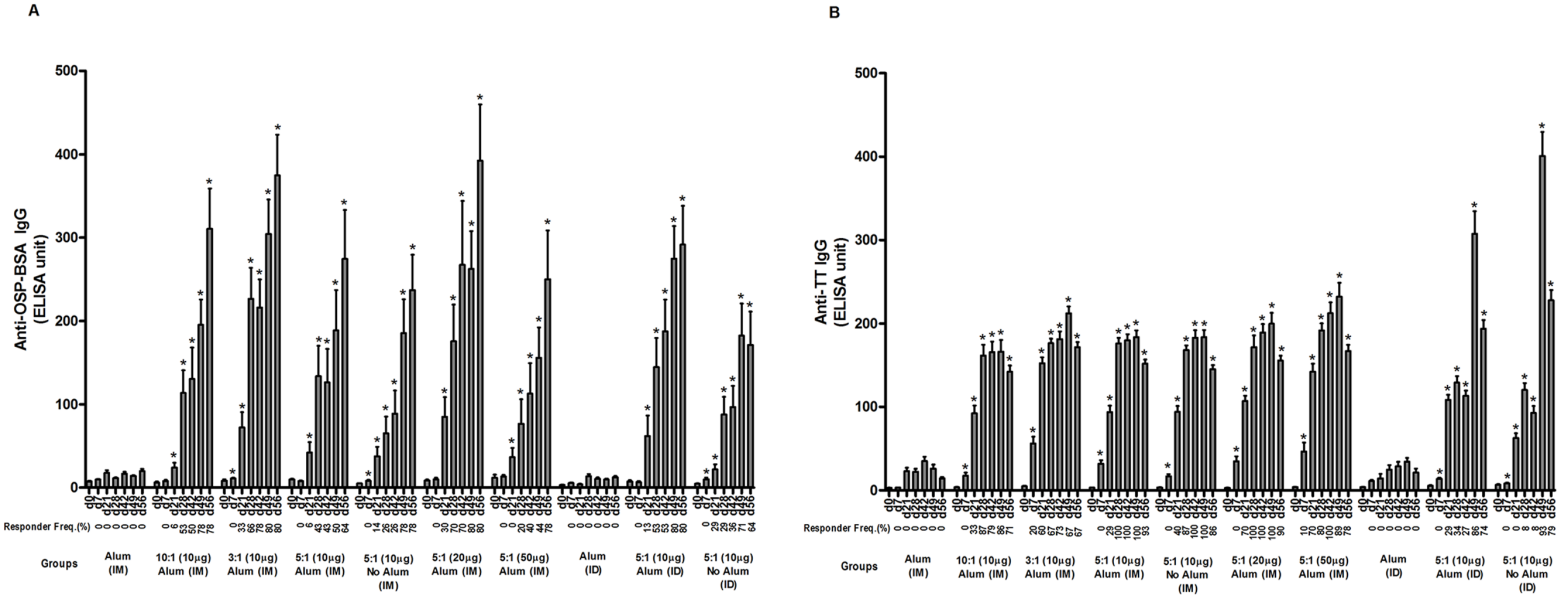
Serum IgG responses at different time points against (A) O-specific polysaccharide (OSP) and (B) tetanus toxoid (TT) in various vaccine cohorts following intramuscular (IM) or intradermal (ID) immunization with various doses (based on OSP component) and/or loading ratios of OSP to TTHc, with or without adjuvant alum. Mice were immunized on days 0, 21, and 42. Responder frequency reflects a 15-fold increase over baseline for anti-OSP IgG, and 30-fold increase for anti-TT IgG. An asterisk denotes a statistically significant difference (*P*<0.05) in the mean response from baseline (day 0) level.

**Fig 4:**
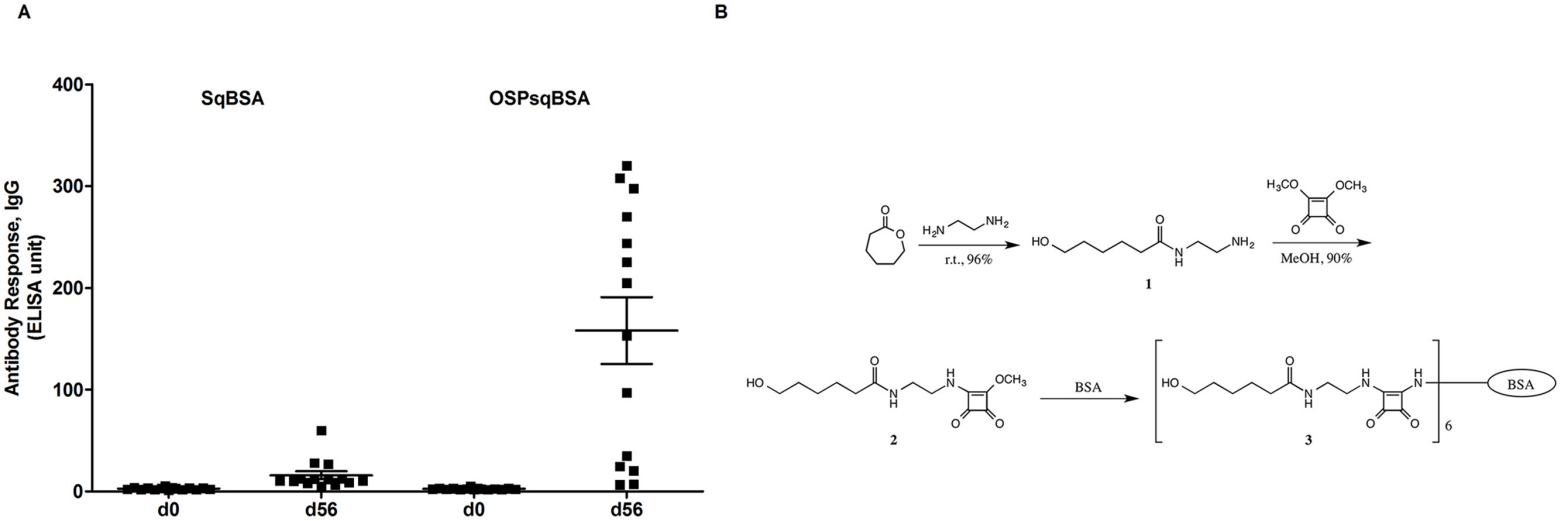
(A) Immune responses against OSP linked to BSA via squarate (ratio ~5:1; OSPsq:BSA) versus an unrelated oligosaccharide linked to BSA via squarate (B; ratio ~6:1; sq:BSA-synthetic conjugate 3, see text) induced by vaccination with alum and OSP linked to rTTHc via squarate (ratio 5:1; OSP:rTTHc). Day 0 and day 56 serum from mice vaccinated with OSP:rTTHc were analyzed using plates coated with squarate-BSA (synthetic conjugate 3; see text) or OSPsq:BSA (OSP:BSA).

**Fig 5:**
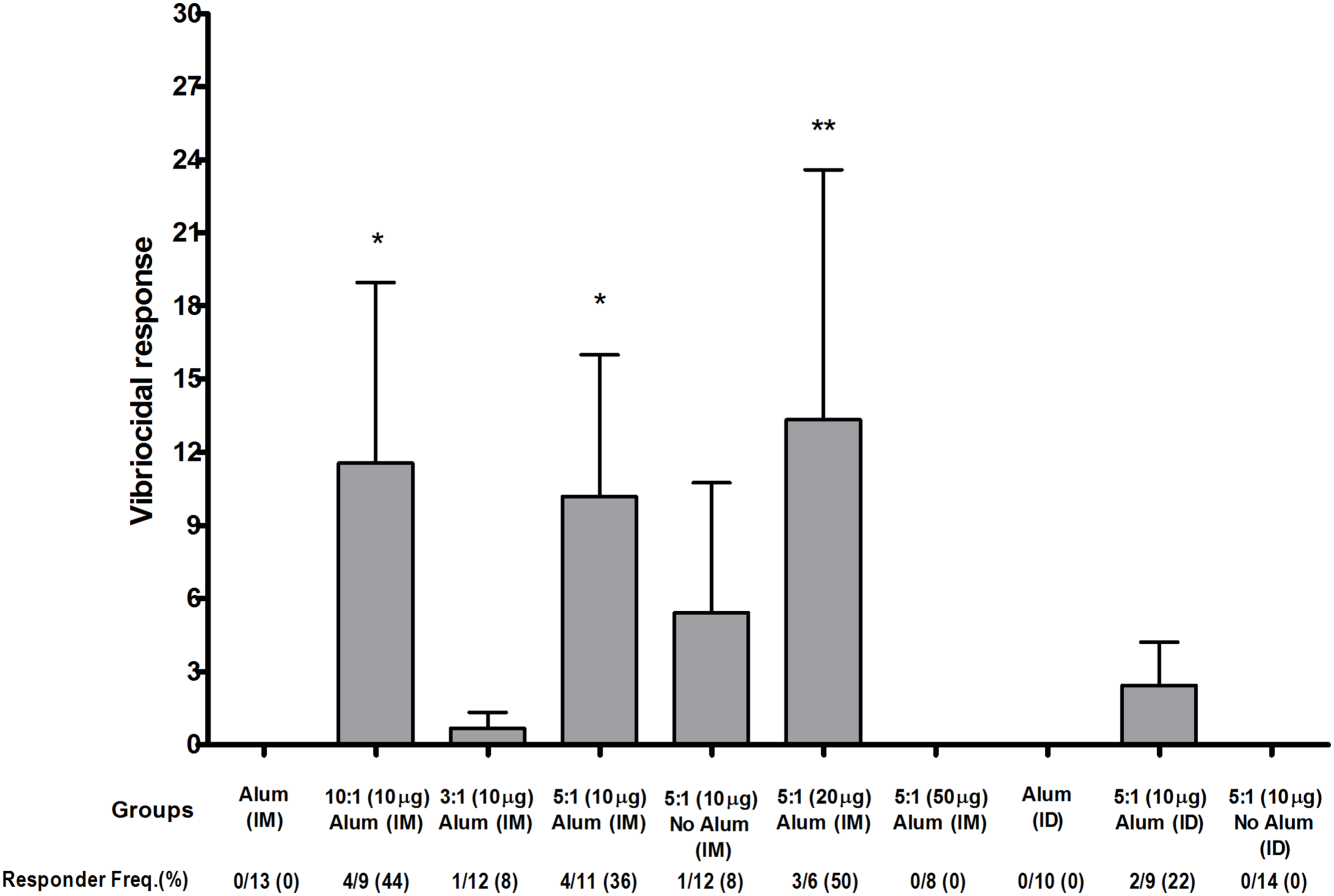
Vibriocidal responses in vaccine cohorts. An asterisk denotes a statistically significant difference (*P*<0.05) in the mean response from the group immunized with only alum.

We found a significant increase in OSP (Fig 3A) and tetanus (Fig 3B)-specific IgG responses in serum of mice who received Inaba OSP-rTTHc vaccine intramuscularly or intradermally. These responses were comparable at different doses and molar ratios of OSP to TThc, with no major increase afforded when the vaccine was administered in the presence of alum. Significant responses (*P*<0.05) against OSP and TTHc were found in all cohorts of mice after a single immunization of vaccine when compared to baseline (day 0) levels, with responses being detectable within 7 days of first immunization in many cohorts. No antigen-specific responses were detected in mice immunized with alum alone. We found no significant increase in serum IgM or IgA responses in any vaccine cohort. In general, immune responses were comparable across all the vaccine cohorts, although anti-OSP responses rose more rapidly in mice immunized with 20 μg of OSP (5:1) compared to responses in mice immunized with 10 μg or 50 μg. Anti-TT responses were highest in mice immunized intradermally, with a significant boosting increase following the third immunization (Fig 3B). Following immunization with OSP:rTTHc, we found no significant induction of antibody targeting the squarate moiety used to attach OSP to carrier (Fig 4). We detected low level vibriocidal responses in vaccinated animals (Fig 5).

### Memory B cell responses

**Fig 6:**
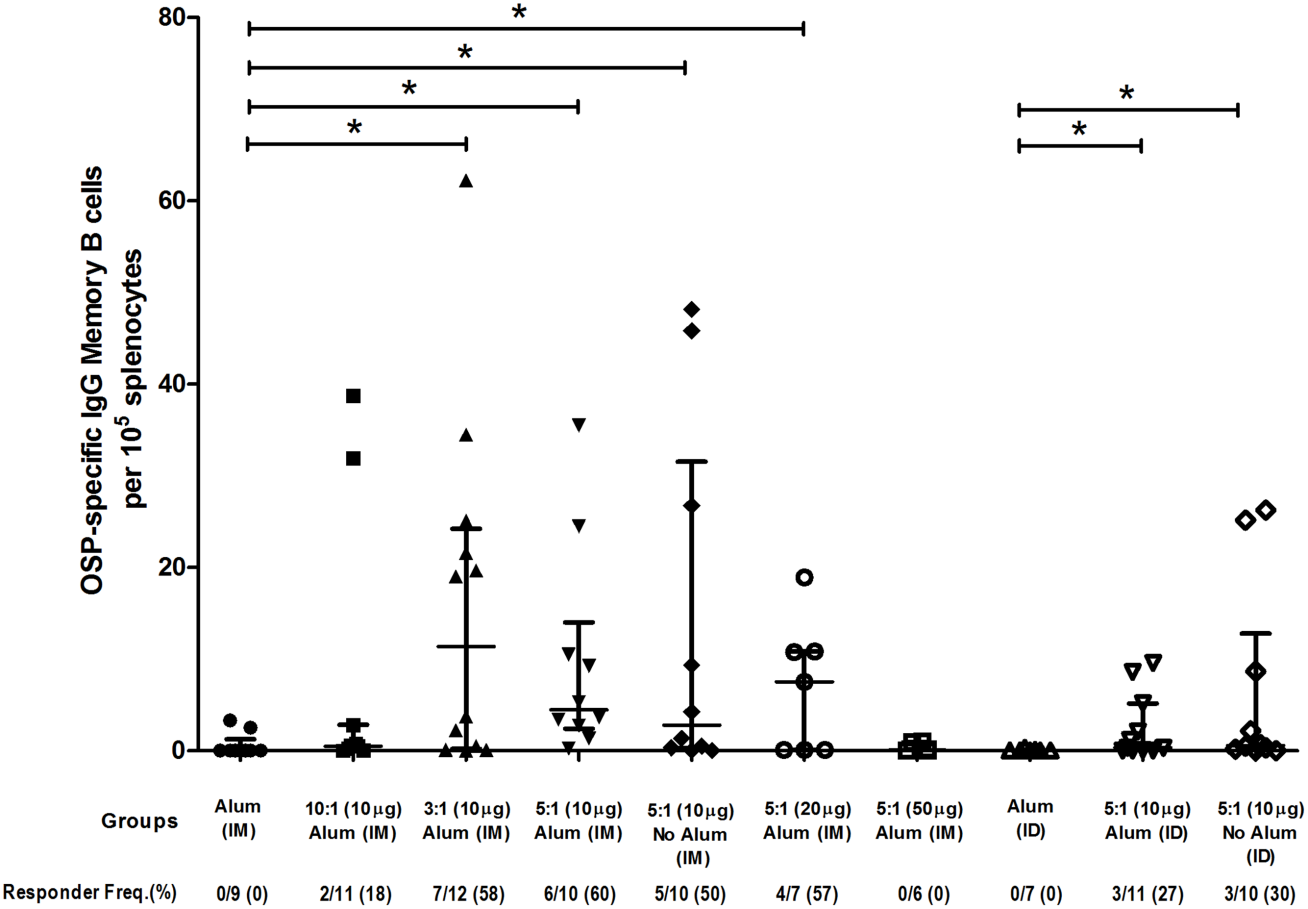
Memory B cell IgG responses in spleen at day 56 targeting O-specific polysaccharide (OSP) in various vaccine cohorts following intramuscular (IM) or intradermal (ID) immunization with various doses (based on OSP component) and/or loading ratios of OSP to TTHc, with or without adjuvantative alum. Mice were immunized on days 0, 21, and 42. Responder frequency reflects an increase of >5 times total IgG secreting cells with stimulation versus no stimulation, and >3.5 anti-OSP spots per 10^5^ splenocytes. An asterisk denotes a statistically significant difference (*P*<0.05) in the mean response from the group immunized with only alum.

Following intramuscular and intradermal immunization, we detected OSP-specific IgG memory B cells in spleen of vaccinated animals at the time of sacrifice (Fig 6) in the various vaccine cohorts, although we did not detect this response in animals receiving the highest dose of vaccine (50 μg of OSP:rTTHc 5:1, intramuscularly). Memory B cells recognizing TT were also detected in the vaccine groups, although low level anti-TT responses were also seen in mice receiving intramuscular alum alone.

### Lamina propria lymphocyte IgA responses

**Fig 7:**
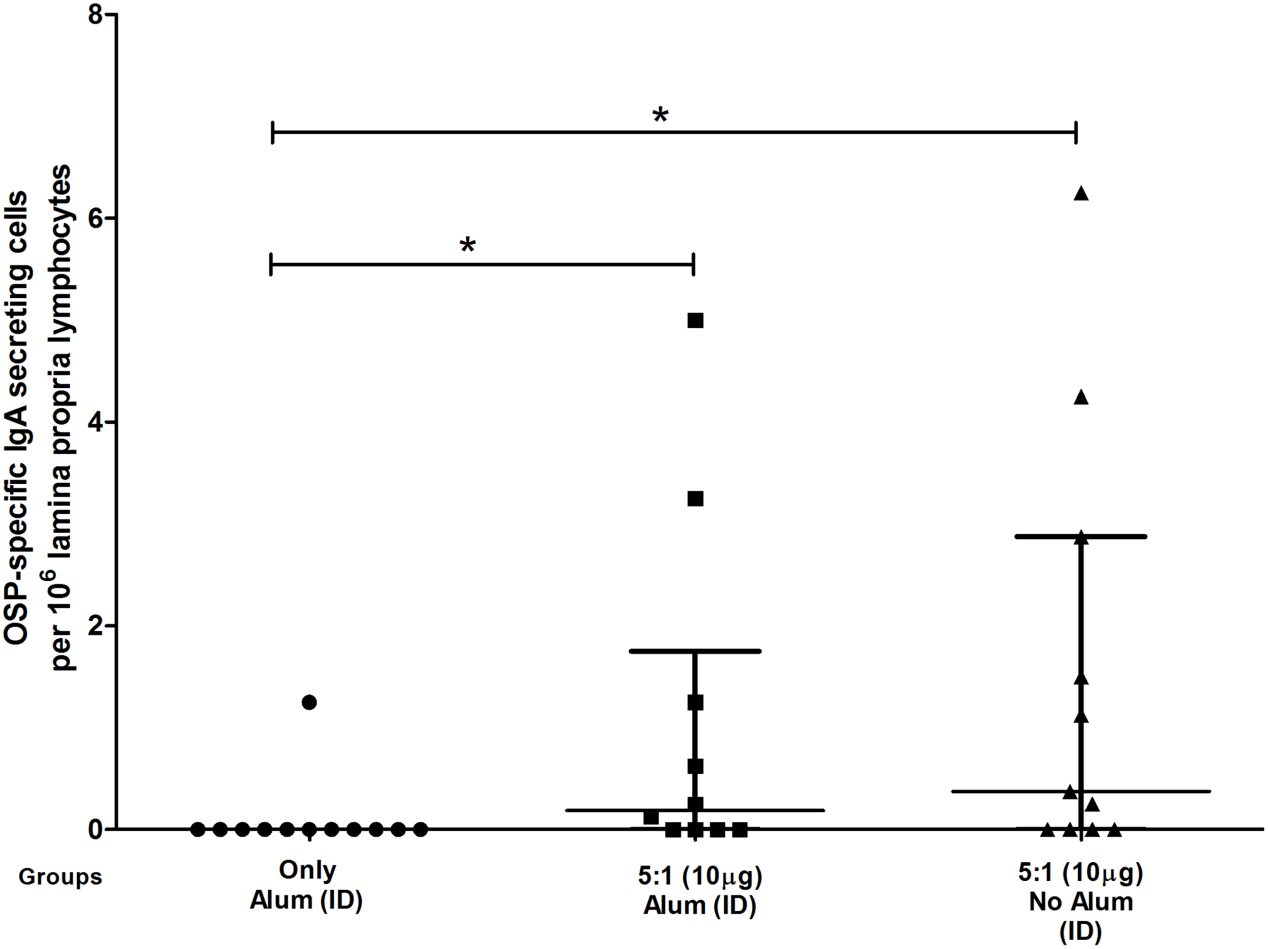
OSP-specific IgA secreting cells in intestinal lamina proprial tissue following intradermal immunization. An asterisk denotes a statistically significant difference (*P*<0.05) in mean from the group immunized with alum only.

We found significant IgA secreting cell responses (*P*<0.05) in the intestinal lamina propria of mice immunized intradermally with OSP:rTTHc in the presence or absence of alum compared with responses in mice receiving alum only (Fig 7). We did not find this response in animals receiving OSP:rTTHc intramuscularly.

### Neonatal challenge

**Fig 8:**
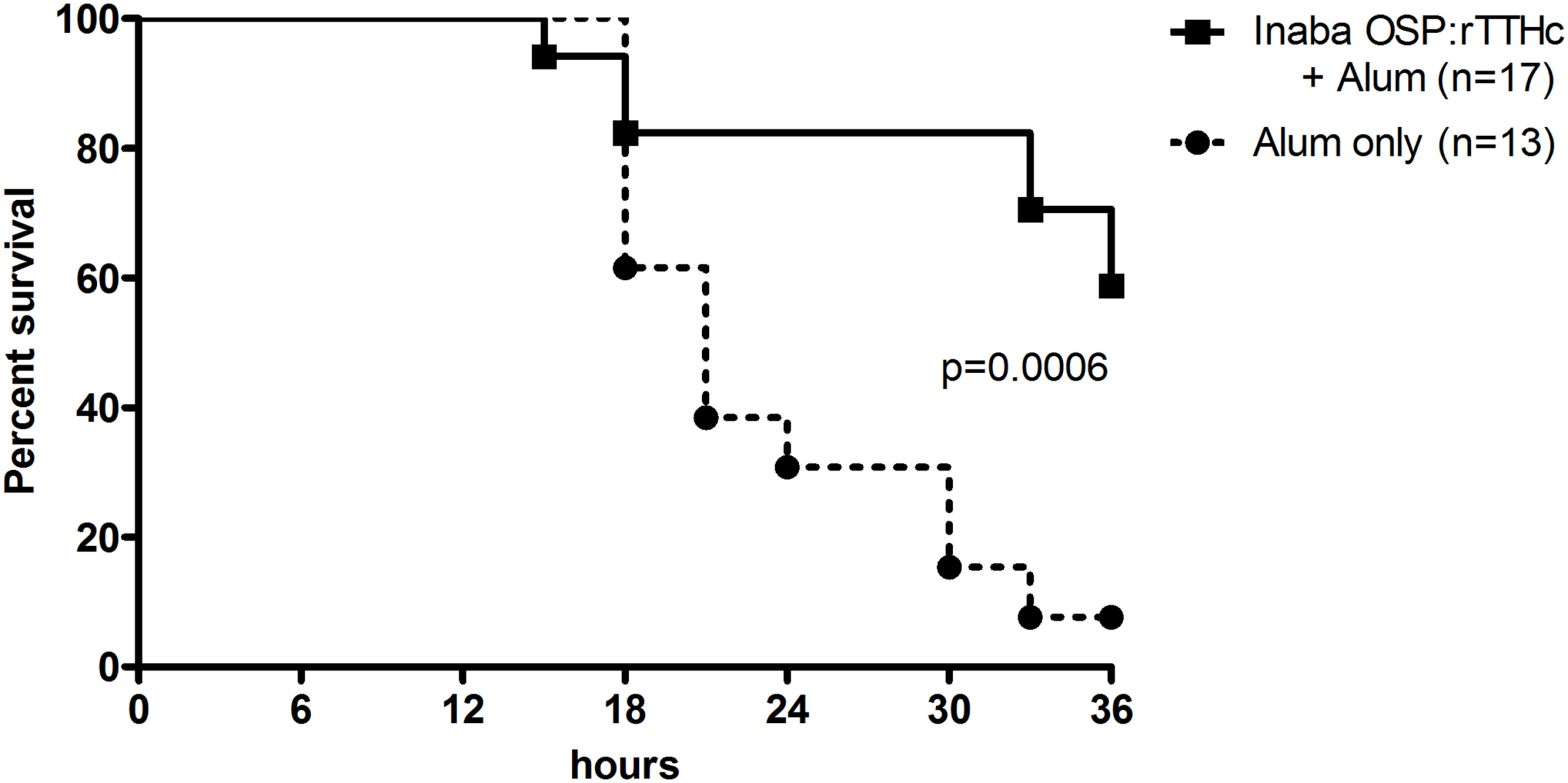
Survival likelihood of neonatal CD -1 mice following oral challenge with wild type *V*. *cholerae* O1 El Tor Inaba strain N16961. Three to five day old pups were orally gavaged with 50 μl of a preparation containing 5.8x10^9^ CFU of wild type *V*. *cholerae* N16961 mixed with a 1:250 dilution of pooled day 42 serum from mice intramuscularly immunized with conjugate vaccine (OSP:rTTHc) and immunoadjuvantative alum, or alum alone. Survival curves were compared by log rank testing.

We found a significant difference in survival between mice challenged with wild-type *V*. *cholerae* O1 Inaba N16961 mixed with sera collected from mice immunized with conjugate and adjuvant (60% survival at 36 hours), compared to mice challenged using sera from mice immunized with adjuvant alone (5% survival at 36 hours; 55% protection; *P*<0.001) (Fig 8).

## Discussion

Immunity against cholera is serogroup-specific and serogroup specificity is determined by the O-specific polysaccharide (OSP) of *V*. *cholerae* lipopolysaccharide (LPS) [9,10,11,16,29,30]. The *V*. *cholerae* O1 serogroup contains both Ogawa and Inaba serotypes [1,31,32]. Currently, the global cholera pandemic is caused by *V*. *cholerae* O1 El Tor organisms with the prevalent serotype shifting between Ogawa and Inaba during cholera outbreaks [33]. We have previously shown that a cholera conjugate vaccine containing *V*. *cholerae* O1 Ogawa OSP is immunogenic in mice, and that responses are enhanced when the vaccine is administered with an ADP-ribosylating immunoadjuvant [16]. Although immune responses across serotypes are cross-reactive, data suggest that previous infection with Ogawa may not provide complete protection against Inaba, while previous infection with Inaba provides more complete protection against both Inaba and Ogawa [13,14,15]. For this reason, we developed a cholera conjugate vaccine containing Inaba OSP from a well-characterized *V*. *cholerae* source strain isolated in 2007 from a patient with cholera in Bangladesh.

To create our conjugates, we used squaric acid chemistry to attach OSP to a carrier protein via single point attachment, resulting in a sun-burst display of OSP to mimic how OSP is presented on the bacterial surface. To confirm that the OSP was displayed in an immunologically relevant manner, we confirmed that OSP was recognized by convalescent phase sera of humans recovering from cholera but not typhoid in Bangladesh. As for the carrier, we used a recombinantly produced immunogenic fragment of tetanus toxoid heavy chain. This protein has been used as a carrier for other conjugate vaccines as well [34].

We next confirmed that the OSP:rTTHc vaccine would be immunogenic, showing that immunization of mice induced prominent anti-OSP and anti-TT serum IgG responses, with significant increases occurring following a single immunization, and additional boosting of immune responses occurring after both second and third immunizations. Overall, we found comparable immune responses in animals immunized with the same quantity of OSP contained within vaccines in varying molar ratios of OSP to rTTHc. Although there was a trend toward higher immune responses in mice immunized with the 3:1 version of the vaccine, it should be noted that these mice received more moles of total vaccine at each immunization compared to mice receiving the 5:1 or 10:1 vaccines. We used the 5:1 version of the vaccine as our central benchmark, and found that co-administering the vaccine with alum did not significantly alter immune responses.

Interestingly, parenteral administration of whole cell cholera vaccine results in protection comparable to that afforded by oral immunization with killed whole cell vaccines, although the previously available parenteral vaccine required multiple administrations, frequent boosters, and was associated with significant local reaction-adverse events [35]. The reasons for the ability of a parenteral vaccine to provide protection against cholera are unclear, although they may relate to the fact that IgG is actively transported across the intestinal epithelial surface in addition to IgM and IgA [36,37,38,39]. Although we were unable to detect circulating antigen-specific IgA responses in the serum of immunized animals, we were able to show that intradermal immunization of OSP:rTTHc induced OSP-specific IgA antibody secreting cells in intestinal lamina proprial tissue. There are very limited data on intradermal administration of conjugate vaccines in general [40], and, to our knowledge, our current study is the first demonstration that intradermal vaccination with a conjugate vaccine can induce anti-saccharide responses in intestinal tissue. We also observed that higher TT-specific responses occurred in mice immunized intradermally compared to those induced in mice receiving the same vaccine dose intramuscularly. These data suggest that intradermal immunization of conjugate may not only induce mucosal responses, but may permit dose reduction [41].

Our conjugation approach uses residual core oligosaccharide effectively as a linker, attaching OSP through core to protein carrier at a single point. This conjugation approach is attractive for a number of reasons: (1) it does not include the additional steps required to introduce a linker, (2) it results in single point sun-burst display of OSP that mimics how OSP is displayed on the bacterium, (3) it can be used to attach a carrier to any OSP that contains a reactive amine group in core oligosaccharide and as such can be used in a platform vaccine development approach, and (4) it is a very simple chemical process that would be readily adoptable for manufacturing purposes in resource-limited areas. In order to directly address whether the squarate moiety itself induced immune response unrelated to OSP or carrier [42,43], we measured anti-squarate immune responses following vaccination with OSP:rTTHc and found no appreciable induction targeting squarate. This observation further supports the use of squaric acid chemistry in development of conjugate vaccines.

The vibriocidal assay measures a functional serum antibody response that correlates imperfectly with protection against cholera [44]. In this assay, vibriocidal antibodies bind complement and lyse *V*. *cholerae in vitro*. There is no vibriocidal value that confidently predicts protection against disease [44,45]. Intestinal IgA does not bind complement, although IgM and IgG do. The vibriocidal response largely targets *V*. *cholerae* LPS [46], and we have recently shown that vibriocidal antibodies largely target OSP [17]. In our current study, we showed that vaccination with OSP:rTTHc resulted in the production of vibriocidal antibodies, although these responses were lower than those normally induced following immunization with currently available oral cholera vaccines [47,48]. This in large measure may reflect that these oral cholera vaccines induce IgM responses that are relatively short-lived [4,44], and IgM antibodies may be more efficient when assessed in the standard vibriocidal assay, since the pentameric structure of IgM compared to IgG may facilitate complement binding or bacterial lysis itself. To further assess the functionality of the antibodies induced by vaccination with OSP:rTTHc, we also showed that serum from immunized mice provided protection against challenge with wild type *V*. *cholerae* in the standard mouse survival model.

Currently available oral cholera vaccines (OCVs) represent a significant advance and are beginning to play an important role in cholera control strategies globally [3]. Two oral cholera vaccines are commercially available internationally. Dukoral (WC-rBS, Crucell, Sweden) contains several biotypes and serotypes of *V*. *cholerae* O1 supplemented with 1 mg per dose of recombinant cholera toxin B subunit. Shanchol (ShanthaBiotechnics-Sanofi Pasteur, India) contains several biotypes and serotypes of *V*. *cholerae* O1 and *V*. *cholerae* O139 without supplemental cholera toxin B subunit. Unfortunately, however, the vaccines are less effective in young children under the age of 5 years compared to protection afforded to older individuals [4,49,50,51]. Dukoral requires three immunizations in children under 5 years of age, with boosters every 6 months [3]. Shanchol may provide longer term protection, but provides only approximately 40% protective efficacy in children under 5 years in a cholera endemic area [52]. When assessed, immunization with an OCV was not associated with induction of memory responses targeting LPS/OSP [4], and in children younger than 5 years of age, its administration was associated with a T-regulatory response, while wild type cholera resulted in a pro-inflammatory response, down-regulation of a regulatory response, and induction of memory responses targeting LPS/OSP [53]. Importantly, the presence of LPS/OSP IgG memory responses correlated with protection against cholera in a prospective study of household contacts of cholera index patients in Bangladesh [54]. In our current study, we show that OSP:rTTHc induces OSP-specific memory responses following intramuscular and intradermal vaccination.

Our study has a number of limitations. We did not assess memory or long lived plasma cell responses in intestinal tissue, we did not assess cross protection against Ogawa challenge, we did not quantify IgG responses in intestinal tissue, and we did not assess protection afforded by intradermal immunization directly. Despite these limitations, we feel that our work is significant since it describes a novel Inaba cholera conjugate vaccine that displays *V*. *cholerae* OSP in an immunologically relevant manner, induces high level anti-OSP responses that are functional and protective, and induces memory responses. We assessed a range of preparations, doses, presence or absence of adjuvant, and immunization routes, and showed that intradermal administration of the vaccine induces OSP-specific responses in intestinal tissue. A cholera conjugate vaccine could have utility as an independent monovalent vaccine, as part of a prime-boost approach when combined with oral cholera vaccination to boost immune responses in young children and to consolidate long term protection, and/or as part of a multivalent conjugate enteric vaccine that targets other intestinal pathogens, such as shigella and salmonella. Importantly, the approach used to produce OSP:rTTHc can be used as a platform technology to generate conjugates containing OSP of other pathogens, linking to recombinant TTHc, and using scalable, characterizable and reproducible processes.
